# Plant and Dairy-Based Yogurts: A Comparison of Consumer Sensory Acceptability Linked to Textural Analysis

**DOI:** 10.3390/foods11030463

**Published:** 2022-02-04

**Authors:** Mitali K. Gupta, Damir D. Torrico, Lydia Ong, Sally L. Gras, Frank R. Dunshea, Jeremy J. Cottrell

**Affiliations:** 1 Faculty of Veterinary and Agricultural Sciences, School of Agriculture and Food, The University of Melbourne, Parkville, VIC 3010, Australia; mitalik@student.unimelb.edu.au (M.K.G.); fdunshea@unimelb.edu.au (F.R.D.); 2 Future Food Hallmark Research Initiative Project, The University of Melbourne, Parkville, VIC 3010, Australia; lon@unimelb.edu.au (L.O.); sgras@unimelb.edu.au (S.L.G.); 3 Department of Wine, Food and Molecular Biosciences, Lincoln University, Lincoln 7647, New Zealand; Damir.Torrico@lincoln.ac.nz; 4 The Bio21 Molecular Science and Biotechnology Institute, Department of Chemical Engineering, The University of Melbourne, Parkville, VIC 3010, Australia; 5 Faculty of Biological Sciences, The University of Leeds, Leeds LS2 9JT, UK

**Keywords:** focus group, microstructure, rheology, gelling, emotions, protein, vegan, dairy substitutes

## Abstract

Yogurt, readily available in plant and dairy-based formulations, is widely consumed and linked with health benefits. This research is aimed to understand the sensory and textural spectrum of commercially available dairy and plant-based yogurts. In a preliminary study, qualitative focus group discussions (4 groups; *n* = 32) were used to determine perceptions of 28 dairy and plant-based yogurts, identifying positive consumer perceptions of plant-based yogurts. A smaller subset of five spoonable and one drinkable yogurts—(Reference, Soy, Coconut, Cookies, Berry, and Drinkable) was subsequently selected for rheological and structural measurements, showing wide variations in the microstructure and rheology of selected yogurt samples. A quantitative blind sensory tasting (*n* = 117) showed varying yogurt acceptability, with Berry being the least-liked and Cookies being the most-liked yogurt, in terms of overall liking. The multi-factor analysis confirmed that compositional and textural elements, including protein content, gel firmness, and consistency coefficient, displayed a positive relationship with overall liking. In contrast, fat, sugar, and calories were negatively correlated to the overall liking. This research showed that texture and other compositional factors are significant determinants of the consumer acceptability of yogurt products and are essential properties to consider in product development.

## 1. Introduction

The world population is set to increase by two billion in the next decade [1] and demand for protein will grow by 20% by 2030 [2]. Therefore, there is a significant interest in developing alternative low-carbon protein sources, such as plant-based yogurts, to fulfill the growing demand and address sustainability issues. The median global warming potential (GWP) for plant-based milk alternatives, such as almond and soy-milk, is lower than that of milk [3] and consuming more sustainable diets can reduce greenhouse gas emissions [4]. Accordingly, plant-based yogurt replacements or substitutes could be more environmentally sustainable [5] and healthier [6]. While there are already established plant-based milk alternatives, the market share of these products of USD 2.2 billion, is only a small percentage of the global milk market, estimated at USD 1.7 trillion [7]. Part of the reason for this smaller market share is the low consumer acceptability of current plant-based dairy-substitutes [8]. Improving the consumer acceptability of milk alternatives, could therefore contribute to a broad strategy for a low carbon future.

Dairy yogurts are a rich source of essential minerals, vitamins and protein, and beneficial bacteria [9]. The lower consumption of plant-based yogurts may be due to their different textural properties, compared to dairy yogurts. The cross-linking of casein proteins is an intrinsic process in the formation of dairy yogurt gels, caused by heating and acidification by bacterial cultures [10], which contributes to the texture of these products. The most common ingredient for plant-based yogurt is soy [11,12], with other plant ingredients, such as coconut and almonds, gaining favor. Novel ingredients, such as lupins [13], oats [14], peas [15], quinoa [16], and flaxseed [17] are also being assessed. The different textural properties of commercial plant-based yogurts could be attributed to lower protein concentrations and the different gelation properties of these proteins compared to casein, requiring the addition of gelling agents. As the texture of the yogurt is a critical component for consumer acceptability, the lack of understanding of plant-based yogurt structure may be a barrier to increased replacement or substitution of dairy-based yogurts. Previous research has shown that some plant-based alternatives can be similarly liked to dairy yogurts in their mouthfeel profile [18]. For example, a recent study of commercial plant-based yogurts (soy, coconut, cashew, almond, and hemp) found that soy and coconut yogurts were identical to dairy yogurts in terms of sensory acceptability and texture [19]. In another study, the probiotic yogurt from soymilk was shown to be comparable to the standard cow milk yoghurt in terms of the physico-chemical attributes [20]. 

Sensory acceptability is affected by compositional and quality factors, such as protein source, texture, fat, sugar, or the form of the yogurt. Consumer liking, in general, positively correlates with the viscosity and smoothness of the product [21]. An improvement in sensory perception was found by using *Lactobacillus rhamnosus* in fermented plant-based coconut, soy, and oat products. [22]. Additionally, a reduction in sugar in strawberry-flavored dairy yogurt positively affected consumers’ purchase intention [23]. Further, the pre-treatment of plant milks with high-pressure homogenization (600 MPa) has shown to produce plant-protein gels with similar viscosity to dairy yogurt after fermentation [24]. Consumer acceptance is also highly dependent on textural factors [25]; hence, it is crucial to link these parameters to the sensory acceptance for yogurt studies. However, studies exploring the link between consumer acceptability of texture, structure, and composition of plant-based yogurts as a product category are limited. 

A standardized approach of a focus group was followed in the present study to select yogurts for a sensory tasting session. The preliminary focus group study helped select yogurts from each of the product categories available commercially including dairy, plant, drinkable and with inclusions, to understand the combination of factors affecting consumer liking. A relationship was further developed between the textural parameters (microstructure and rheology) and sensory attributes (taste, odor, appearance, liking, mouthfeel, emotions, and product attributes) to understand the drivers of liking and consumer acceptability for yogurt products. 

## 2. Materials and Methods

### 2.1. Experimental Overview 

This experiment comprised three separate components:

(a) Consumer-guided selection of available yogurt formulations using perceptual mapping (Section 2.2).
(b) Rheological and microstructural analysis of test dairy and plant formulations identified in (a) (Section 2.3).
(c) Blind consumer sensory analysis comparing dairy yogurts with the plant-based formulations identified in (a) (Section 2.4).

Human perceptual mapping and sensory analysis were approved by the Human Ethics Committee of Faculty of Veterinary and Agricultural Sciences (FVAS), University of Melbourne, Australia (Ethics ID 1545786.2 and 1853507.2). The inclusion criteria were that participants consumed yogurt, did not have allergies to wheat, peanuts, added sulphites, and tree nuts and were not lactose intolerant. Participants were provided with a gift voucher as an incentive for participation in the study.

### 2.2. Selection of Test Formulations by Perceptual Mapping

Due to the large amount of dairy and plant-based formulations commercially available, perceptual mapping was used on consumer focus groups to refine the selection of six formulations for subsequent analysis. Perceptual mapping consists of facilitated sessions where consumers are provided a range of yogurt sensory attributes (taste, aroma, texture, and mouthfeel), emotions, and preferred time of consumption. Furthermore, the consumers categorize their assessment on a perceptual X-Y map [26].

#### 2.2.1. Product Classification and Stimuli Selection

A sample of 28 representative yogurts was selected from the Australian market for the study to test a range of products and reflect the stimuli design of the experiment. Products were categorized into four major groupings based on their characteristics (Table S1, Supplementary Data). These were: plain dairy spoonable yogurts without any inclusions (collectively termed as plain dairy); yogurts with differing consistency ranging from spoonable, drinkable or in the form of chunks (collectively referred to as differing consistency); yogurts with additives including fruit or high protein (collectively referred to as additives); and plant-based yogurts based on almond, soy, or coconut (collectively termed as plant-based). The samples were presented to the participants labelled with a three-digit code. They tasted the products and described the sensory attributes.

#### 2.2.2. Focus Group Panels and Facilitation

A staged, consumer-guided and facilitator-led qualitative approach of perceptual mapping [26] was followed for the selection of the test formulations for further testing. In stage 1, an informed consumer panel (*n* = 8), working in the dairy field and were a part of the research project, tasted (gustatory) and visually evaluated 28 yogurts overall (in groups of two), and ranked these according to perceptual mapping technique (Section 2.2.3). They short-listed a set of 16 yogurt products for further consumer testing in stage 2 with untrained consumers, who consumed yogurt at least weekly. The testing was conducted in four focus group studies (total participants, *n* = 32) of 1 h duration. Consumers in each group tasted (gustatory and visually evaluated) the 16 test yogurts and were divided into two segments, Western (*n* = 16) and Asian (*n* = 16), to look for cultural differences in yogurt consumption using perceptual mapping. Consumers were asked to self-identify themselves as Westerns or Asians, and separate panels were conducted for each cultural group. Consumption insights from stages 1 and 2 were used for the final test sample selection (Section 2.2.5).

An experienced facilitator ran all consumer sessions. Each session began with introducing the researchers and participants (5 min), followed by understanding familiarity with yogurt, frequency of consumption, and eating occasion (15 min). Further, product and descriptor mapping were conducted for the tasted yogurts (30 min). The session ended with a general discussion on consumption and preference of yogurts (10 min).

#### 2.2.3. Perceptual Mapping 

Consumers in each group tasted the stimulus yogurts and were asked to place these on a pre-determined X-Y map according to resemblance or contrast. The X-axis ranged from sour (left) to sweet (right) and Y-axis from healthy (top) to indulgent (bottom), as labeled by the facilitator. These axes were chosen based on the inherent characteristics of yogurt, which is sweet or sour and representing the perception of the yogurt, based on healthy or indulgent. The facilitator placed the first sample at the center of the plot in each session, which was a plain dairy yogurt and was asked to be taken as a Reference by the consumer panels. They then placed each of these yogurts on an X-Y perceptual map as a group. The perceptual mapping exercise was to understand how consumers assess product attributes, based on which most of the insights were generated. Replicate sessions were carried out to validate the generated insights [27]. 

#### 2.2.4. Yogurt Descriptor Mapping

The focus group panels were presented with a random list of ‘emotions’ and ‘sensory attributes’ printed on a sheet (Table S2). These descriptors included a list of general terms used to classify food items and discussed the likability of sensory attributes (taste, aroma, texture, and mouthfeel) and emotions (positive, negative, and neutral terms). Participants were asked to select (as a group) the top terms they would use to describe the tasted yogurt in each session or could use their words. The facilitator and note-taker recorded these terms. 

#### 2.2.5. Analysis of Results, Selection of Top Descriptors and Test Formulations

The research team analyzed the notes at the end of each focus group session. Observations were discussed, and insights were generated across both ethnic groups. Six test yogurts were selected from 16 yogurt formulations for further analysis based upon consumer insights from perceptual mapping and covering the four major groupings of the sensory space (Table 1). The test yogurts selected were a plain dairy Greek yogurt (reference), coconut and soy-based yogurts. Furthermore, dairy-based yogurts containing berries, chocolate biscuit, and drinkable yogurt were included to establish a diverse range of consumer responses to textures and high likability responses [28,29]. Top-rated descriptors were also selected based on consumer insights. The emotion terms were divided into positive, negative, and neutral, whereas sensory attributes represented taste, aroma, texture, and mouthfeel, as shown in Table 2.

### 2.3. Functional Properties of Yogurt Samples

#### 2.3.1. Rheological and Textural Properties 

A rheometer (Discovery HR-2 Hybrid rheometer, TA Instruments, New Castle, DE, USA) equipped with a cone plate (40 mm diameter/2° angle), was used to measure the apparent viscosity (η) with varying shear rate (s^−1^) from 0.1 s^−1^ to 100 s^−1^, for each of the six yogurt samples in Table 1. The flow behavior index (n) and consistency index (K) were calculated by fitting the data to the modified power-law (η = K γ^n−1^), using the rheology software TRIOS v4.2.1.36612 (TA Instruments, New Castle, DE, USA). A pre-shear of 100 s^−1^ for 10 s was applied to each sample, prior to measurements. All measurements were performed in triplicate for each sample at 10 °C, which was also the serving temperature for sensory analysis. A texture analyzer (TA.HD plus, Stable Microsystems, New Castle, DE, USA) was used to measure gel firmness with a 5 kg load cell, using a trigger force of 1 g and a 10 mm cylindrical probe with each sample analyzed five times [30]. 

#### 2.3.2. Microstructure Analysis

Confocal laser scanning microscopy (CLSM) was used to observe the microstructure of the six yogurt samples in Table 1 using an established protocol [31]. A small amount of sample from the middle part of the product container was placed on a microscope slide in between a spacer (2 mm in thickness and 10 mm in diameter). The yogurt samples were stained with Fast Green FCF and Nile Red, after ten-fold dilution of stock solutions (1 mg/mL) with purified water. After removing the excess stain, a glass coverslip (0.17 mm thick; ProSciTech, QLD, Townsville, Australia) was put on top of the spacer, in contact with the sample. Samples were observed using an inverted confocal scanning laser microscope (Leica SP8; Leica Microsystems, Heidelberg, Baden-Wurttemberg, Germany) with a ×63 oil-immersion objective. The excitation/ emission wavelengths were set at 488 nm/500–600 nm for Nile Red and 633 nm/ 650–710 nm for Fast Green FCF. A total of 9 images were collected for each sample at different magnifications and representative images are shown for each sample. 

### 2.4. Quantitative Sensory Analysis

#### 2.4.1. Participants

In a blind tasting test using sensory booths, participants were asked to taste and answer questions relating to each of the six yogurt samples in Table 1. Questions included the hedonic ratings of attributes such as odor, appearance, taste, overall liking, and mouthfeel of the product tasted. Samples were fully randomized. The consumers’ demographic data were also collected (*n* = 117, age: 20–68 years, gender: 42 males and 75 females, ethnicity: 73 Asians and 44 Westerns) and is compiled in Table S3 (Supplementary Data).

#### 2.4.2. Samples

All the six products used for tasting were commercially available in Australian supermarkets. A 10–15 g sample was served in a small, covered container labeled with 3-digit random numbers and each sample was given to the sensory panel one at a time in a randomized order. All participants were given instructions about the tasting in a briefing room before they were seated in individual booths for tasting. 

#### 2.4.3. Data Collection

Participants were asked to taste the sample and answer the questions related to each product on the screen in front of them. Sensory data were recorded on Samsung Galaxy View 18″ tablets (Seoul, South Korea) using the Bio-Sensory app (University of Melbourne, Melbourne, VIC, Australia) for Android Tablet PCs. All the sensory tests were conducted in a sensory facility located in the Faculty of Veterinary and Agricultural Sciences at the University of Melbourne, Australia. Sensory booths were illuminated with white LED lights. The temperature of the room was controlled to 22–24 °C, and the yogurt was served at a temperature of 8–10 °C. Participants were asked to rate the appearance, odor, taste, and overall liking of the sample on a continuous 0–15 points scale [32]. This was later scaled down to 1–9 points hedonic scale [33], where 1—dislike extremely, 5—neither dislike nor like, and 9—like extremely. The 9-point hedonic scale is the standard measuring tool for acceptability tests found in the literature. The mouthfeel (or thickness) of the samples was rated on the Just About Right (JAR) 5-point scale, which was scaled down to 3, where 1—too thin, 2—just about right (JAR), and 3—too thick [34]. Consumers were also asked to select all the emotion terms and sensory attributes that they could link to the tasted product, from the list provided in Table 2, using the check-all-that-apply (CATA) method [35]. Emotion words and sensory attributes were shortlisted from the focus group studies (marketplace insights) by the untrained groups. The emotion terms were divided into positive, negative, and neutral and sensory attributes represented taste, aroma, texture, and mouthfeel. A group of 12–15 participants were allotted specific time slots to attend the tastings. They were seated in a briefing room and were given instructions for tasting the samples. They were then taken to individual booths where the samples were served with each participant given 15–20 min for the tastings.

### 2.5. Statistical Analysis

Minitab software (version 19.1, Minitab Inc., State College, PA, USA) was used for the analysis of the quantitative sensory and rheological data. The difference of means was assessed by one-way analysis of means (ANOVA) and Fisher’s LSD comparison using a significance level of *p* < 0.05. The best subsets regression model and the general linear model were used to correlate the emotion and attribute terms with the overall liking scores. Multi-factor analysis, conducted to create a link between the sensory and textural parameters in yogurts and penalty analysis, for mean drops of mouthfeel ratings, were performed using XLSTAT (by Addinsoft, New York, NY, USA, version 2020.1.1).

## 3. Results

### 3.1. Insights Generated from Preliminary Qualitative Study

**Figure 1:**
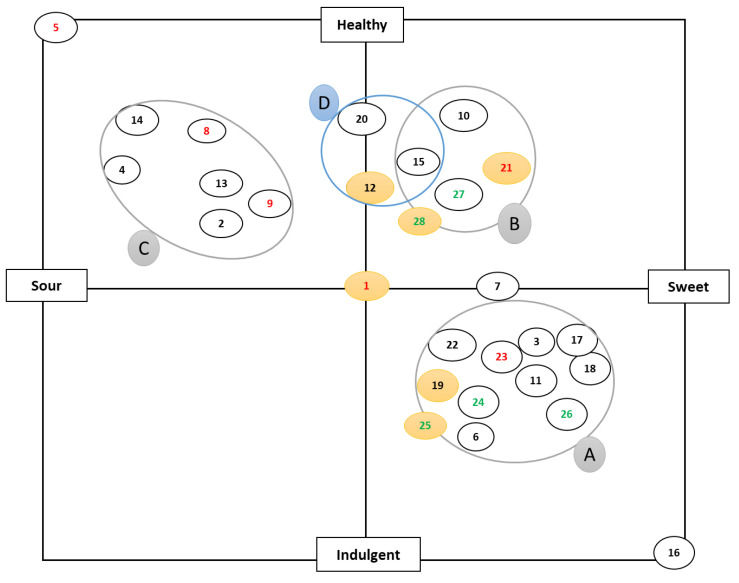
Perceptual map classified into three major groups of yogurts, group A—Treat or dessert, group B—Mid-morning snack, group C—Breakfast, and group D—Drinkable yogurts (popular specifically among Asians). Product codes are as given in Table S1 (Supplementary Data). The figure is representative of the perceptual map for 28 yogurts (Section 2.2.1) that covers all the initial yogurt samples, marked with a number 1 to 28 in black, red, or green. Specific products highlighted in yellow are representative of the samples selected for further testing, the yogurts marked in red letters have a protein content >6%, the yogurts marked in green letters are plant-based yogurts.

The focus group discussions in the qualitative study identified the twenty-eight yogurt products into four different groups on the perceptual X-Y map (Figure 1) according to time of consumption. Different ethnicities (Westerns and Asians) preferred to consume yogurts differently, as shown by the groupings; three consumption part of day groupings were identified for both the cultures, including yogurt to be consumed as (Group A) treat or dessert, (Group B) as a mid-morning snack, and (Group C) as breakfast. Another (Group D) drinkable group was specific to Asians. These concepts were developed based on the interpretations of all focus group studies:

(a) Group A (Treat or dessert): The market has a high number of these products. These are sweet and indulgent products containing added sugar, fruits, and other flavorings.
(b) Group B (Mid-morning snack): These are typically yogurts without any flavors or fruits, labeled with perceived health benefits, such as low sugar, low fat, lactose-free or probiotics.
(c) Group C (Breakfast): A strong sour after-taste of the yogurt is considered an attribute that reinforces the perception of healthiness. These typically require an additional component (e.g., cereal or fruit) for consumption to mitigate the sour after-taste, resulting in a ritual type of behaviour.
(d) Group D (Drinkable): An additional group, specific to Asian consumers, was identified for consumption after dinner or before bed.

Plant-based yogurt alternatives also fitted in all three groupings, except drinkable. Drinkable plant-based yogurt would be a new space to try, as it could not be found in the market space when the study was conducted. Plant-based yogurts were ranked in between the healthy and indulgent anchors of the scale for both types of consumers, who were not very familiar with their taste. In this study, focus group discussions found consumers were also looking for health imparting properties, such as probiotics, omega-3 fatty acids, products that are easy to digest, a healthy snack or a dessert. The focus groups were broadly divided based on ethnicity into Western or Asian cultural groups, as the different cultures have varied consumption habits. Western consumers preferred yogurt with inclusions, such as cereals or like to add their own fruit. In contrast, Asians preferred light yogurt and were more familiar with a drinkable format, consistent with a prior study by Sifferlin [28]. 

The yogurts marked in red letters have a protein content greater than 6%. These yogurts were spread across the X-Y plot, showing that the protein concentration was not related to any specific day part groupings. The products highlighted in yellow were selected for further testing. Product 1 was selected to represent the sour and healthy section of the X-Y plot, as suggested by consumer insights. The plant-based yogurt products 25 and 28 (soy and coconut, respectively) being unsweetened plain versions, were selected to eliminate any bias during the tasting sessions. Soy and Coconut yogurts were selected for further testing based on the higher preference of these two types compared to Almond yogurt. Savoury yogurts mainly covered the X-Y plot between sour and indulgent, but these were considered dips by the sensory panellists, hence not included in the study. Furthermore, product 12 was replaced with another Asian drinking yogurt (product 11) for tasting, as that product was no longer locally available at the time of study.

### 3.2. Perceptual Mapping of Yogurts

Texture and taste played an important role in the discrimination of products in the transcripts and perceptual maps, while protein content and type did not affect the positioning, as observed for participants of both Western and Asian origins (Figure 1). Added inclusions in yogurts, such as fruits or cereals, affected the consumer preference of different cultural groups. Furthermore, products with differing consistency also affected liking, as in the case of the drinkable types, especially for Asian consumers. The participants reported that plant-based yogurts (containing a different protein type) were both “novel” and “sustainable” in focus group discussions.

### 3.3. Differences in Yogurt Composition, Microstructure and Texture

The composition of the six yogurt products used for more detailed analysis, as listed on product labels, is shown in Table 3. Composition varied widely between the products. Protein varied from 9.7 g in the Cookies product to 0.7 g in the Coconut product. Sugar varied from 16 g in the Berry product to 1 g in the Coconut product and fat was highest at 11 g in the Coconut product and lowest at 1.2 g in the Cookies product. All products contained thickeners or stabilizers, except for the Reference and Drinkable products. 

**Figure 2:**
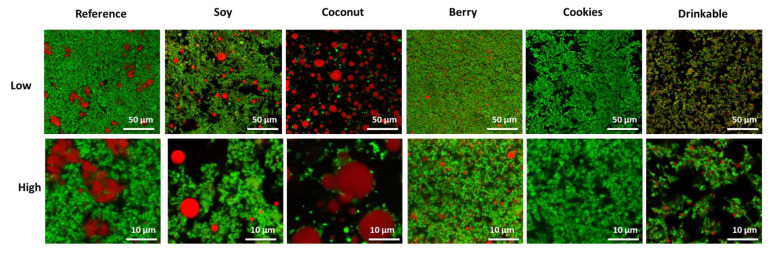
The microstructure of the six yogurt samples. Representative images are shown for the Reference, Soy, Coconut, Berry, Cookies, and Drinkable yogurts, labelled at the top of each column; see Table 3 for corresponding sample composition. The fat appears red in these images and the protein appears green, black unstained areas are the serum pores and other unstained ingredients including carbohydrate and stabilizers. Lower magnification images appear in the upper row, where the scale bars are 50 µm in length and higher magnification images of the same samples appear in the bottom row, where the scale bars are 10 µm in length.

The microstructure of the yogurt samples varied considerably (Figure 2); this included large differences in the size, shape and integration of the fat, differences in the extent of protein aggregation and network formation, and differences in unstained regions, which can include stabilizers and thickeners. This range of structures was anticipated, in part, as a result of differences in composition (Table 3). The different structures are also expected to contribute to product texture and sensory perception.

The Cookies type low-fat yogurt (1.5 g fat; Table 3) contained little fat visible within the protein network. Fat was more visible in the other samples, particularly in the high fat Coconut yogurt (11 g fat; Table 3), which contained large fat globules ~10 µm in diameter that were the dominant component of the yogurt, with no protein matrix and a large non-stained serum phase containing starch. The fat droplets were still relatively large in the Reference (~3 µm) and Soy yogurts (~5 µm). The fat in the Berry (~1 µm) and Drinkable samples (~1 µm) was smaller and appears more integrated within the protein network, possibly due to high shear homogenization. Homogenization of milk before fermentation reduces the size of the milk fat globule, increasing interactions with the milk protein. The protein-coated fat globules participate in network formation, as the pH of the milk reduces during fermentation, contributing to the firmness of the yogurt get [30]. The protein network in the Drinkable yogurt also appears less continuous, due to the additional shear potentially employed after the fermentation process, as is common in the production of drinkable yogurt [36]. 

Large unstained areas, possibly occupied by starch or stabilizers, could be observed in the Soy, Coconut, and Cookies yogurt products. However, the stabilizers appear well-integrated into the network of the Berry yogurt product. The different concentrations of stabilizers and processing, including heat treatment and shear, may also have led to differences observed in the microstructure of these yogurt products.

**Figure 3:**
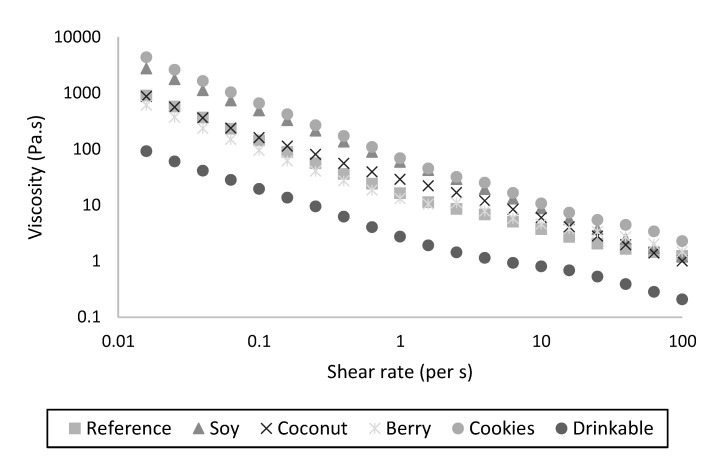
Viscosity of the six yogurt products shown in Figure 2, as a function of shear rate.

All five spoonable yogurts (Reference, Soy, Coconut, Berry and Cookies) had a higher viscosity than the Drinkable sample across the range of shear rates tested. A range of viscosities was observed, each resulting in a reasonably similar viscosity profile as a function of shear rate, as shown in Figure 3. The two plant-based spoonable products (Soy and Coconut) differed slightly in their viscosity profile; the Soy product was most similar to the Cookies dairy product, while the Coconut product was most similar to the Reference and Berry dairy products, with subtle differences at shear rates higher than 10 s^−1^. 

**Figure 4:**
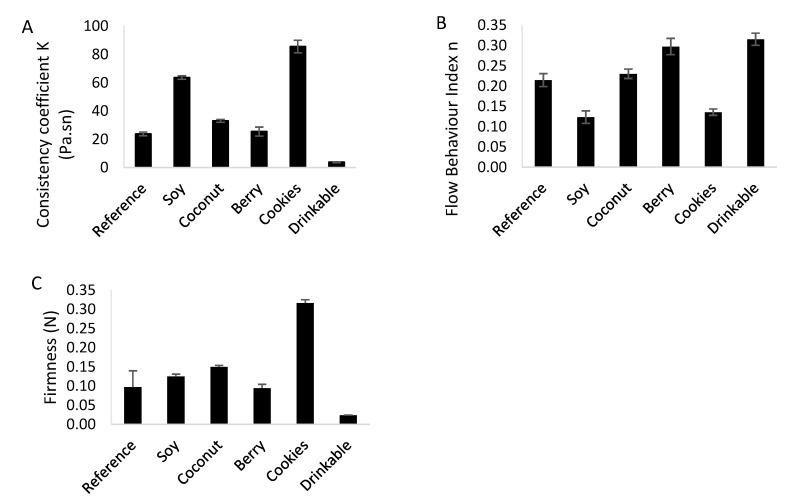
The rheological properties of the six yogurt products shown in Figure 2. (**A**) flow behavior index (n), (**B**) consistency coefficient (K), and (**C**) gel firmness, were determined for each sample. Standard error bars represent standard deviation of the means.

The viscosity data in Figure 3 were used to calculate the consistency coefficient (K) and flow behavior index by fitting to the power law (Figure 4A–C). The flow behavior index (n) values for all yogurts were less than 1 (Figure 4B), indicating shear thinning behavior, with the Soy and Cookies products showing the lowest n and highest deviation from Newtonian behavior. The highest consistency coefficient value (K) was for the Cookies product (Figure 4A), indicating a higher firmness, consistent with the higher gel firmness for this yogurt (Figure 4C). This product had both high protein content and stabilizers, seen in the microstructure (Figure 2), which contribute to the high firmness.

### 3.4. Differences in Acceptability of the Samples

In the quantitative tasting experiment, where the yogurts were served blindly (using 3-digit random codes for all samples), the demographic factors including age, gender, and ethnicity did not influence the overall liking scores for each attribute (*p* > 0.05). Therefore, populations were pooled for the subsequent quantitative analysis. In terms of odor and appearance acceptability (Table 4), as given by the consumers on the 9-point hedonic scale, all the samples had similar likings, except for Coconut, which had the lowest liking score for all the measured attributes. The overall liking and taste liking were closely related, and the Cookies yogurt was the most-liked product, followed by the Drinkable yogurt, then the three plain yogurts (Reference, Soy, and Coconut) and the Berry product with the lowest acceptability score. Reference and Soy yogurts were rated similarly (*p* > 0.05) for appearance, taste, and overall liking. 

### 3.5. Effect of Overall Liking on Mouthfeel

Penalty analysis was carried out for the mouthfeel rating, which followed a similar trend as for the overall liking. The Cookies yogurt had the highest frequency of just-about-right in mouthfeel or thickness. The Drinkable yogurt was rated “too thin”, and the Berry yogurt was rated “too thick” (Table 5). The drinkable yogurt had entirely different properties and cannot be compared on the same scale as the spoonable yogurts. The liking of Drinkable yogurt was relatively high, even if the JAR (just-about-right) values on the firmness were low compared to other samples. Comparing the mean drops for the products, Soy and Berry had non-significant (*p* > 0.05) penalties, which suggested that the texture was “just-about-right” for these products. In the case of the Reference product, 67% of participants rated this to have the right texture (JAR). For the penalty analysis, a *p* < 0.05 indicates that the overall liking of this sample was negatively affected by the mouthfeel texture (either “too thin” or “too thick”). Overall liking scores of Berry and Soy were not affected significantly by the texture. In the case of Berry, there can be two possible reasons for this effect. Firstly, the acceptability of this product was too low (Table 6), so mouthfeel did not affect it in terms of the magnitude of the difference. Secondly, the flavor was the main driver in disliking this product, whereas, Soy had a better mouthfeel, as suggested by its liking score.

### 3.6. Effect of Overall Liking on Emotions and Product Attributes

A linear model was developed to link the emotional words and sensory attributes with the overall liking of the product. A general linear model was developed, showing an R^2^ value of 78.6%. Emotional terms were selected based on their significance (*p* < 0.05) (Table 6). Overall liking was dependent on the terms related to texture, as rated by consumers. The attribute terms “good texture”, “creamy”, “smooth” and “light” were positively linked to the overall liking (*p* < 0.05) and “bad texture” was negatively linked with the overall liking (*p* < 0.05). Additionally, the emotional terms “cheerful”, “neutral” and “trusted” were positively linked to the overall liking, whereas “nasty”, “indifferent” and “artificial” were negatively linked to the overall liking (*p* < 0.05).

### 3.7. Comparing Rheology with Sensory Properties

**Figure 5:**
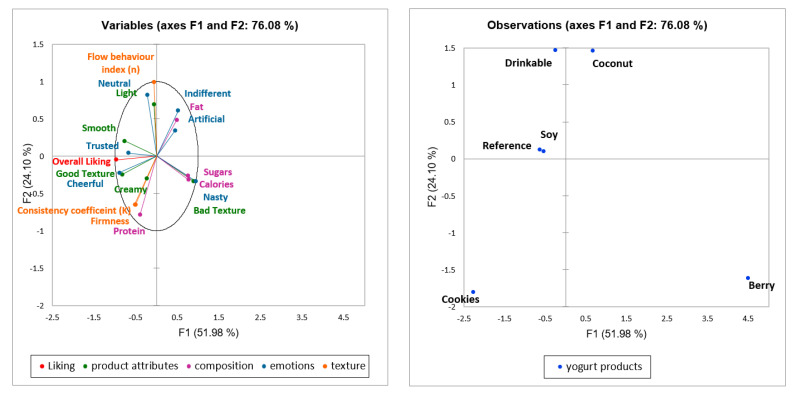
Multi-factor analysis plot showing a relationship between overall liking with the functional attributes (composition and texture) and sensorial attributes (emotions and product attributes) for different yogurt products with 76.08% variation.

While linked to the textural and compositional factors, overall liking was also linked to emotion terms and product attributes, as rated by consumers. A multi-factor analysis (MFA) approach was used to develop a link between yogurt sensorial and functional parameters (Figure 5). Axis F1 represents 52.0% variability and F2 represents 24.1% variability, overall explaining 76.08% variation for the overall data (see Supplementary Table S4). High sugar and calories were related to “bad texture” and “nasty” emotions with factor loadings between 0.7 to 1.2 on PC1, these functional attributes were inversely linked to overall liking and were linked to Berry yogurt. High fat was related to the product being “artificial” and “indifferent”, with factor loadings between −0.1 to −0.6 on PC2 and was related to Coconut yogurt. An opposite response was observed with the functional attributes protein content, gel firmness, consistency coefficient (K) and a “creamy” emotion, with factor loadings between −0.4 to −0.8 on PC1, as related to product Cookies, which is also the highest liked yogurt product. The overall liking was related to “cheerful” and “trusted” emotion terms and, also to “good texture” and “smooth” product attributes, as shown by Reference and Soy yogurts, which were closely related on PC1 with factor loadings between −0.8 to −0.1. Drinkable yogurt was rated to be “light” and “neutral”, with factor loadings between −0.3 to −0.5 on PC2.

## 4. Discussion

### 4.1. Sensory Attributes

Consumers show differences in the selection of foods based on their culture [37]. Both qualitative and quantitative sensory techniques were used to understand the effect of culture on yogurt selection. The initial qualitative study helped to short-list the six products for further testing, based on the factors that affected consumer likability, including, protein type (dairy or plant) and content, yogurt form (drinkable or spoonable), and added inclusions (fruit or cereal). While the qualitative analysis showed differences in consumption and liking behaviors of participants towards yogurts, the quantitative analysis did not show significant differences (*p* > 0.05) for the interactions between the overall liking and the demographic factors, including ethnicity, age group, gender, and consumption frequency. A similar effect was observed in a study with sheep-meat, where quantitative tests found no significant differences between ethnicities [26]. However, focus groups were successfully used as preliminary tests to understand consumer liking and select samples for further quantitative testing [38].

Yogurts with cereals and fruits are considered healthy meals [39]. In the present quantitative sensory study, the Cookies yogurt received the highest score for the overall liking (in the quantitative sensory study, Table 4), followed by the Drinkable yogurt. The product Cookies was the most-liked yogurt, possibly due to the perceived additional health benefits due to the presence of cereal type particles [40]. The drinkable yogurt also had high liking, despite having the lowest gel firmness and consistency coefficient (Figure 4), consistent with the recent increased popularity of drinkable yogurts [41].

Berry received the lowest overall liking, even though it had a continuous dense protein network structure in which homogenized fat particles were integrated (Figure 2). High sugar decreased overall liking, as consumers are becoming conscious about sugar intake, which is linked to perceptions of lowered healthiness. These explanations were determined by the unsolicited feedback provided by participants after the tasting of the six yogurts and are consistent with prior work where sugar reduction in strawberry-flavored yogurt positively affected consumers’ purchase intention [23].

Texture majorly affected product liking and was the most crucial factor for spoonable yogurts, as seen by the relationship of “good texture” and “smooth” terms with “cheerful” emotion, whereas “bad texture” term with “nasty” emotion. Textural terms, such as “creamy”, “body” and “viscosity” had a positive correlation with the acceptability of semi-solid desserts in another study [42]. Additionally, the most frequently used terms for describing the liking of the desserts were “thick”, “soft” and “yummy” [43]. In another study on milk desserts, consumers positively linked textural terms such as “thick”, “creamy” and “nice” to textural liking and negatively related “liquid”, “bad texture” and “not much creamy” to texture liking [44].

### 4.2. Functional Attributes

The products tested in this study were quite different in terms of composition, microstructure, and rheological properties, key factors impacting on consumer liking. Previously, others have also found yogurt microstructure to help understand sensory acceptability [45]. 

The Reference and Soy yogurts were rated similarly in terms of the overall liking by consumers (Table 4), consistent with prior studies [19], and had a similar microstructure (Figure 2), giving rise to a similar gel firmness (Figure 4) and only slightly different rheological properties. This finding indicates that the type of protein (dairy or plant) was not the major deciding factor for liking the yogurts. Interestingly, the Reference yogurt selected in this study was not typical of a dairy yogurt, which often features a homogeneous protein network with homogenized fat droplets integrated throughout the network, as shown in the study by Nguyen, Ong, Lefèvre, Kentish and Gras [31]. Instead, the reference dairy yogurt examined here had large, coalesced fat droplets non-homogeneously distributed in the protein network, likely as a result of differences in the production process. Significantly, the Soy yogurt had a better texture than the Coconut yogurt, where the gel consisted of fat particles in a starch matrix, lacking any protein network (Figure 2).

Overall liking was positively related to protein content. High protein provided a better gel firmness and higher consistency coefficient (K), as seen by the Cookies yogurt, increasing the overall liking scores. Overall liking was negatively related to sugar, fat, and calorie content. In a study of calorie-reduced dairy products, low-calorie dairy was a preferred motivator of product choice [46]. Consumers consider low sugar content to be linked to lower calorie intake [47], although this isn’t always the case, leading to preferences for either no-added or reduced sugar products [48]. Thickeners and stabilizers were also present in the plant-based yogurts, although their presence didn’t appear to reduce the overall liking in Soy. Morell et al. (2015) [49] reported increased liking in yogurts including starches due to higher creaminess and thicker consistency. In this study, two factors were found important: the concentration of thickener and stabilizer affects likeability, as the Coconut yogurt with a starch matrix was not highly liked, the integration of thickeners and stabilizers into the protein network is also critical, as seems in the well-liked Cookies and Soy products.

Viscosity increased the product liking, as seen for the spoonable yogurts. The Cookies yogurt, which had the highest viscosity, was the most-liked yogurt. This is consistent with another study that showed a positive correlation between consumer liking, viscosity, and smoothness [21]. Viscosity can also increase the satiating capacity of yogurt, regardless of the presence of other particles [50]. In a study on Greek yogurts, consumers liked yogurts with a firmer texture [51]. In contrast, the matrix in the drinkable yogurts is broken due to the shearing action [36]. In this study, the effects of viscosity on liking were inconsistent for the Drinkable yogurt, which had a lower viscosity but still showed a higher overall liking compared to other products. 

### 4.3. Relationship between the Sensory and Textural Attributes

A combination of factors, including composition and texture, affect sensory attributes and liking scores for yogurt products. A strong correlation has previously been observed between composition and rheological factors and overall liking for oat-based gels served as dairy alternatives, where key drivers of liking were related to sweet, moist, soft, and smooth descriptors, as rated by consumers [52]. The ideal profile of strawberry yogurt has also been found to have intermediate smoothness and viscosity and low levels of fruit particles and acid taste [21], through consideration of textural and sensory attributes. Liking is also affected by consumers’ expectations [53], as confirmed in the MFA plot in this study (Figure 5), where “cheerful” and “trusted” emotion terms, and also “creamy”, “smooth” and “good texture” attributes were used by participants. Textural properties have previously been found to be important to the acceptance of natural yogurts, although sensory texture was less critical compared to other factors such as off-flavor and bitterness [54]. Other studies have also found dairy yogurts and their plant-based alternatives were similarly appreciated in terms of their mouthfeel profile [18], consistent with the findings of this study.

## 5. Conclusions

There is an increasing trend to include more sustainable plant-based protein sources as dairy alternatives for yogurts. However, more research is needed to overcome non-optimal product properties, including the microstructure and texture, which are important for sensory properties and product development. Compositional factors, such as sugar, calorie and protein content also influence product success, affecting liking. Consumer rated emotions and product attributes can explain more about the liking of a yoghurt, and further help in creating elaborate models to develop links with texture and compositional attributes. When considering novel protein sources, a product development strategy is likely to be most successful if it incorporates an understanding of the structural and textural product parameters, ensures the protein source best fits the design of the yogurt, and links these properties to consumer liking.

## 6. Limitations and Future Research

More plant-based alternatives, with inclusions and added ingredients in varying concentrations, can be further tested to understand the liking scores and consumer acceptability of these products. Rather than commercial samples, laboratory-made products made in similar conditions can be a better indicator of the exact consumer preferences. Moreover, it would be good to understand consumer preferences and expectations of plant-based yogurts compared to dairy yogurts, to understand further consumer expectations from this novel product category and what measures can be undertaken to make these products more popular alternatives.

## Figures and Tables

**Table 1:** Description of the yogurts selected for further quantitative sensory, microstructural, and rheological analyses.

Product Code	Yogurt Type
Reference	Dairy (plain) − reference sample
Soy	Plant − soy (plain)
Coconut	Plant − coconut (plain)
Berry	Dairy + berry (sweet)
Cookies	Dairy + crunchy (sweet)
Drinkable	Dairy drinkable (sweet)

Yogurt products mentioned in the table above are represented as # in Supplementary Table S1.

**Table 2:** Product descriptors representing the factor and factor type for the yogurt category selected using the perceptual mapping technique.

Factor	Factor Type	Descriptor
Sensory Attribute	Taste	Sweet, Sour, Aftertaste
	Aroma	Mild aroma, Strong aroma
	Texture	Thick, Runny, Lumpy, Good texture, Bad texture, Heavy, Light
	Mouthfeel	Creamy, Smooth, Grainy, Chalky
Sensory Emotion	Positive	Cheerful, Luxury, Trusted, Uplifting, Dependable
	Neutral	Neutral, Guilt-free, Basic, Indifferent, Common
	Negative	Nasty, Deceitful, Cheap, Artificial, Pretentious

**Table 3:** Composition of yogurt products, as listed on compositional labels (per 100 g of product).

Product Code	Protein (in g)	Fat (in g)	Sugars (in g)	Calories (in KJ)	Thickeners/Stabilizers
Reference	8.7	4	2.7	366	None
Soy	4.9	3.9	1.7	285	Hydroxypropyl distarch phosphate, Guar gum
Coconut	0.7	11	1	501	Native starch
Berry	5.9	5.7	16	607	Hydroxypropyl distarch phosphate, Xanthan gum
Cookies	9.7	1.2	2.9	386	Locust bean gum
Drinkable	3.7	2.4	7.7	302	None

**Table 4:** Mean scores ± standard deviation of products for overall liking, appearance rating, odor rating, and taste rating by consumers.

Product Code	Overall Liking	Appearance Liking	Odor Liking	Taste Liking
Reference	5.10 ± 2.27 ^c^	6.34 ± 1.76 ^b^	5.88 ± 1.67 ^c^	4.91 ± 2.34 ^c^
Soy	5.35 ± 2.11 ^c^	6.22 ± 1.87 ^b^	6.37 ± 1.84 ^ab^	5.37 ± 2.11 ^c^
Coconut	4.36 ± 1.99 ^d^	5.70 ± 1.95 ^c^	5.98 ± 1.53 ^bc^	4.27 ± 2.17 ^d^
Berry	1.89 ± 1.04 ^e^	3.44 ± 1.76 ^e^	3.49 ± 1.75 ^d^	1.81 ± 0.98 ^e^
Cookies	7.20 ± 1.50 ^a^	6.90 ± 1.60 ^a^	6.45 ± 1.70 ^a^	7.26 ± 1.50 ^a^
Drinkable	5.90 ± 2.05 ^b^	5.06 ± 2.01 ^d^	5.59 ± 1.89 ^c^	6.21 ± 1.98 ^b^

^a,b,c,d,e^ Means with different superscripts in each column indicate significant differences (*p* < 0.05) by the Fischer’s Least Square Difference test.

**Table 5:** Penalty analysis showing the ‘just-about-right (JAR)’ percentages representing mouthfeel for each of the yogurt products.

Product Code	Level	Percentage Consumers	Mean Drops	Penalties	Mean (Overall Liking)	*p*-Value
	Too Thin	16%	1.7		4.0	
Reference	JAR	67%		1.8	5.7	<0.01
	Too thick	17%	1.9		3.8	
	Too Thin	26%	0.5		5.1	
Soy	JAR	65%		1.7	5.6	0.09
	Too thick	9.4%	1.2		4.4	
	Too Thin	17.1%	2.2		3.1	
Coconut	JAR	46%		1.6	5.2	<0.01
	Too thick	37%	1.3		3.9	
	Too Thin	20%	0.1		1.9	
Berry	JAR	27%		0.1	2.0	0.59
	Too thick	53%	0.1		1.8	
	Too Thin	11%	1.3		6.0	
Cookies	JAR	79%		0.8	7.4	0.03
	Too thick	9.4%	0.1		7.3	
	Too Thin	70%	2.0		5.3	
Drinkable	JAR	29%		2.0	7.3	<0.01
	Too thick	0.9%	3.7		3.6	

**Table 6:** Emotion and attribute variables closely related to the overall liking for the yogurt products, as ranked by consumers.

Factors	Type	Variables	Means		Confidence Interval (95%)		*p*-Value
			Factor 1	Factor 0	Low Level	High Level	
Emotions	Positive	cheerful	5.70	4.26	1.14	1.75	<0.01
	Neutral	neutral	5.23	4.72	0.20	0.82	<0.01
	Negative	nasty	4.25	5.71	−1.81	−1.11	<0.01
	Positive	trusted	5.45	4.50	0.57	1.33	<0.01
	Neutral	indifferent	4.69	5.27	−0.96	−0.20	<0.01
	Negative	artificial	4.62	5.34	−0.97	−0.46	<0.01
Product Attributes	texture	good texture	5.26	4.70	0.28	0.83	<0.01
	texture	bad texture	4.76	5.19	−0.78	−0.08	0.01
	texture	creamy	5.12	4.84	0.02	0.53	0.03
	texture	smooth	5.13	4.82	0.06	0.56	0.02
	texture	light	5.12	4.83	0.02	0.56	0.04

Only significant terms (*p* < 0.05) for emotions and product attributes are presented in the linear model.

## Data Availability

The datasets generated for this study are available on request to the corresponding author.

## Supplementary Materials

The following supporting information can be downloaded at: https://www.mdpi.com/article/10.3390/foods11030463/s1, Table S1: The categorization of yogurts in four categories, which were used for the shortlisting of products for the untrained tasting panels. Table S2: Random list of ‘emotions’ and ‘sensory attributes’ presented to the consumers in focus group discussions. Table S3: Demographic characteristics of the consumers (*n* = 117). Table S4: Factor loadings for multi-factor analysis for a combination of liking, product attributes, emotions, composition, and texture variables.
