# Supplementary material for: Plant and Dairy-Based Yogurts: A Comparison of Consumer Sensory Acceptability Linked to Textural Analysis
Source: Foods. 2022 Feb 4;11(3):463. doi: 10.3390/foods11030463 (PMC8834546; doi:10.3390/foods11030463)
Supplement: Supplementary file 1 [file foods-11-00463-s001.zip › foods-1539724-supplementary/foods-1539724-supplementary.pdf]

Supplementary Material:

Table S1. The categorization of yogurts in four categories, which were used for the shortlisting of products for the untrained tasting panels.

Product Category	Sub-category	Brand Name	Product Name	Nutritional Info (per 100 g)				Product Code
				Protein (in g)	Fat, Total (in g)	Calories (in KJ)	Carbohydrates, Sugars (in g)	
Plain Dairy	Cow	Chobani	Plain whole milk (Greek)	8.7	4	366	2.7	1*#
		Jalna	organic - pot set	4.5	5.4	89	2.9	2*
		Tamar Valley	Greek style	5.3	9.8	542	5.2	3
	Goat	Caprilac	Goat Yoghurt	4.2	4.3	430	6.3	4
	Sheep	Meredith Dairy	Sheep Yoghurt	6.6	7.1	434	3.2	5*
Differing Consistency	Spoonable	Pauls	Farmhouse Gold - vanilla	3.7	5.5	469	11.7	6
		Milk Thief	Kefir Yoghurt - vanilla					7*
		Rokeby Farms	Quark Yoghurt	10	0.7	275	4.2	8
		Woolworths	Skyr	9.6	<0.5	254	2.9	9*
		Liddells	Lactose free - plain	4.5	2.9	354	9	10
	Drinkable	Purejoy	Original	3.7	2.4	302	7.7	11#
		Yili Group	Ambpoerial					12*
		Farmers Union	Greek style - Pouch	5.2	3.3	314	6.1	13
		The collective dairy	Kefir 13	3.7	2	225	3.8	14*
		Yoplait	Petit Miam - Vanilla	3.4	2.3	305	7.6	15*
	Chunks/ Bars	Go Natural	Berry Frugo's	2.2	25.4	2120	64.2	16
Additives	Flavored/ with Fruits	Danone	Activia -Vanilla	4.9	3.8	329	5.1	17
		Dairy Farmers	Thick & creamy - Vanilla	3.7	6.3	525	12.9	18*
		Gippsland	Blueberry	5.9	5.7	607	15.9	19*#
		Vaalia	my first yogurt - vanilla	4.7	2.8	330	7.3	20*
	Crunchies	Chobani	Fit - Vanilla cookie crush	9.7	1.2	386	2.9	21*#
	Grains	Chobani	Oats - Blueberry	7.3	0.6	363	9.4	22
	High protein	Danone	YoPro+ (15 g) - vanilla	9.5	0.3	243	3.2	23*
Plant-based	Coconut	Cocobella	Coconut Yoghurt	0.9	9.7	541	2	24
		Nakula	Unsweetened	0.7	11	501	1	25*#
	Almond	Impressed	Vanilla flavor	1.4	4.1	381	5	26*
	Soy	Soy Life	Soy yogurt	3.3	0.7	330	10.1	27
		Kingland	Soy yogurt	4.9	3.9	285	1.7	28*#

**Yogurt products were subsequently selected as the 16 samples for untrained consumer panels.*

#Yogurt products selected for further quantitative sensory, microstructural, and rheological analyses (as mentioned in Table 1).

Table S2. Random list of ‘emotions’ and ‘sensory attributes’ presented to the consumers in focus group discussions

Mouthfeel	Texture	Flavor/ Aroma	Emotion terms	
inclusions	grainy	acidic	addictive	joyful
creamy	firm	tangy	adult	light
dessert/ ice cream	thin	sour	adventurous	luxury
pleasing/ luscious	thick	salty	aggressive	mellow
artificial	creamy	milky	arrogant	mild
runny	cheesy	not milky	artificial	modern
thick	mayo	neutral	aspirational	nasty
light	lumpy	delicate	basic	natural
clean	bad/ low	light	boring	neutral
good	medium	fruity	brave	nostalgic
smooth	good/ high	very sweet	candid	ordinary
not smooth	strong contrast	sweet	charming	outgoing
not natural	oily	slight sweet	cheap	outrageous
lacking	fatty	not sweet	cheeky	premium
powdery	soft	low on flavor	cheerful	pretentious
frothy	smooth	sugar aroma	cheese-cake	pure
standard	not smooth	mild	child-like	quality
unique	crumbly	strong	clean	quiet
inconsistent	gel	no yoghurt taste	common	quirky
not well mixed	fluid	savory	confident	reckless
watery	watery	clean	confused	refreshing
oily	crunchy	bland	conservative	relaxed
lumpy	powdery	complex	crazy	rich
grainy	sticks to mouth	balanced	creative	satisfying
chalky	drinking type	not balanced	curious	silly
	whipped	vinegary	deceitful	sincere
	light	lemony	delicate	sociable
	heavy	minty	dependable	sour power
		fatty	disappointing	special
		makes mouth water	easy-going	specific
		creamy after-taste	energetic	stimulating
		oily after-taste	exciting	subtle
		after-taste	feminine	surprising
		sweet after-taste	formal	traditional
		oily after-taste	guilt-free	trusted
		after-taste	handmade	unique
		sweet after-taste	healthy	uplifting
			honest	vegan
			imaginative	wild
			indifferent	wrong
			indulgent	youthful
			interesting	

Table S3. Demographic characteristics of the consumers (n = 117)

Characteristics	Category	Number of Consumers
**Gender**	Female	75
	Male	42
**Age**	20-29 years	51
	30-39 years	36
	>40 years	30
**Ethnicity**	Western	44
	Asian	73
**Diet**	Vegetarian	20
	Not on a specific diet	107
**Yogurt Consumption Frequency**	Daily	27
	2-3 times a week	37
	Weekly	33
	Monthly	20

Table S4. Factor loadings for multi-factor analysis for a combination of liking, product attributes, emotions, composition and texture variables

Variables	F1 (PC1)	F2 (PC2)
**Overall Liking**	-0.970	-0.047
**Gel firmness**	-0.502	-0.649
**Consistency coefficient (k)**	-0.524	-0.647
**Good Texture**	-0.060	0.990
**Bad Texture**	-0.250	-0.074
**Creamy**	1.298	-0.488
**Smooth**	-0.079	-0.101
**Light**	-0.125	0.033
**Cheerful**	-0.039	0.457
**Neutral**	-0.741	-0.184
**Nasty**	-0.087	0.328
**Trusted**	1.170	-0.417
**Indifferent**	-0.566	0.034
**Artificial**	0.236	0.280
**Protein**	0.173	0.132
**Fat**	-0.390	-0.784
**Calories**	0.492	0.488
**Sugars**	0.749	-0.266
